# On a new genus and species of karst-dwelling freshwater crab (Crustacea, Brachyura, Potamidae) from Peninsular Malaysia

**DOI:** 10.3897/zookeys.1277.181453

**Published:** 2026-04-09

**Authors:** Zhi Wan Tan, Qie Ooi, Peter K. L. Ng

**Affiliations:** 1 Department of Biological Sciences, Faculty of Science, National University of Singapore, 16 Science Drive 4, Singapore 117558, Singapore Asian Institute of Medicine, Science and Technology University Bedong Malaysia; 2 Asian Institute of Medicine, Science and Technology University, Jalan Bedong—Semeling, 08100 Bedong, Kedah, Malaysia Department of Biological Sciences, Faculty of Science, National University of Singapore Singapore Singapore; 3 Lee Kong Chian Natural History Museum, Faculty of Science, National University of Singapore, 2 Conservatory Drive, Singapore 117377, Singapore Lee Kong Chian Natural History Museum, Faculty of Science, National University of Singapore Singapore Singapore

**Keywords:** Cave, karst, limestone, Potamiscinae, taxonomy

## Abstract

Recent explorations of caves in Pahang, central Peninsular Malaysia yielded specimens of an unusual long-legged terrestrial, cave-dwelling crab. Morphological examinations of the specimens collected, in comparison with known species from the region suggests that they belong to a new genus and species, which are described herein. *Merapohra karsticola***gen. nov. et sp. nov**., is differentiated from other potamids by characters of the carapace epigastric and postorbital cristae, ambulatory legs, male thoracic sternum, male pleon, and diagnostic structure of the male first gonopod. The discovery of this new genus and species of cave-dwelling crab highlights the importance and high diversity of karst systems in Peninsular Malaysia, which are imperilled in light of ongoing quarrying and mining activities.

## Introduction

The primary freshwater crab fauna of Peninsular Malaysia is relatively well studied, with numerous taxonomic studies and various scientific expeditions conducted over the years (Roux 1936; Bott 1966; Ng and Tan 1984; Ng 1985, 1986a, 1986b, 1988, 1989, 1990a, 1990b, 1992a, 1992b, 1993, 1995, 1997, 2017, 2018a, 2020; Ng and Yang 1985; Ng and Lim 1986; Ng and Ng 1987; Ng and Tan 1991; Ng and Takeda 1992; Yeo et al. 1999; Yeo 2001; Ng and Lee 2012; Ng and Schubart 2014; Ng and Ahmad 2016). There are currently 51 species known from Peninsular Malaysia, belonging to two families: Potamidae Ortmann, 1896 and Gecarcinucidae Rathbun, 1904 (Ng and Yeo 2007; Ng and Lee 2012; Ng and Schubart 2014; Ng and Ahmad 2016; Ng 2017, 2018a, 2020; Ng and Ng 2026). The majority of species belong to the family Potamidae, with 33 species currently recognised, assigned to four genera: *Baccazia* Ng, 2018; *Gempala* Ng & Ahmad, 2016; *Johora* Bott, 1966; and *Stoliczia* Bott, 1966. Most potamid crab species in Peninsular Malaysia are generally associated with cool, fast flowing streams and waterfalls (Ng 1988, 2020), or have a more terrestrial habit, often associated with montane moist cool habitats (Ng and Schubart 2014; Ng 2018a).

**Figure 1.**
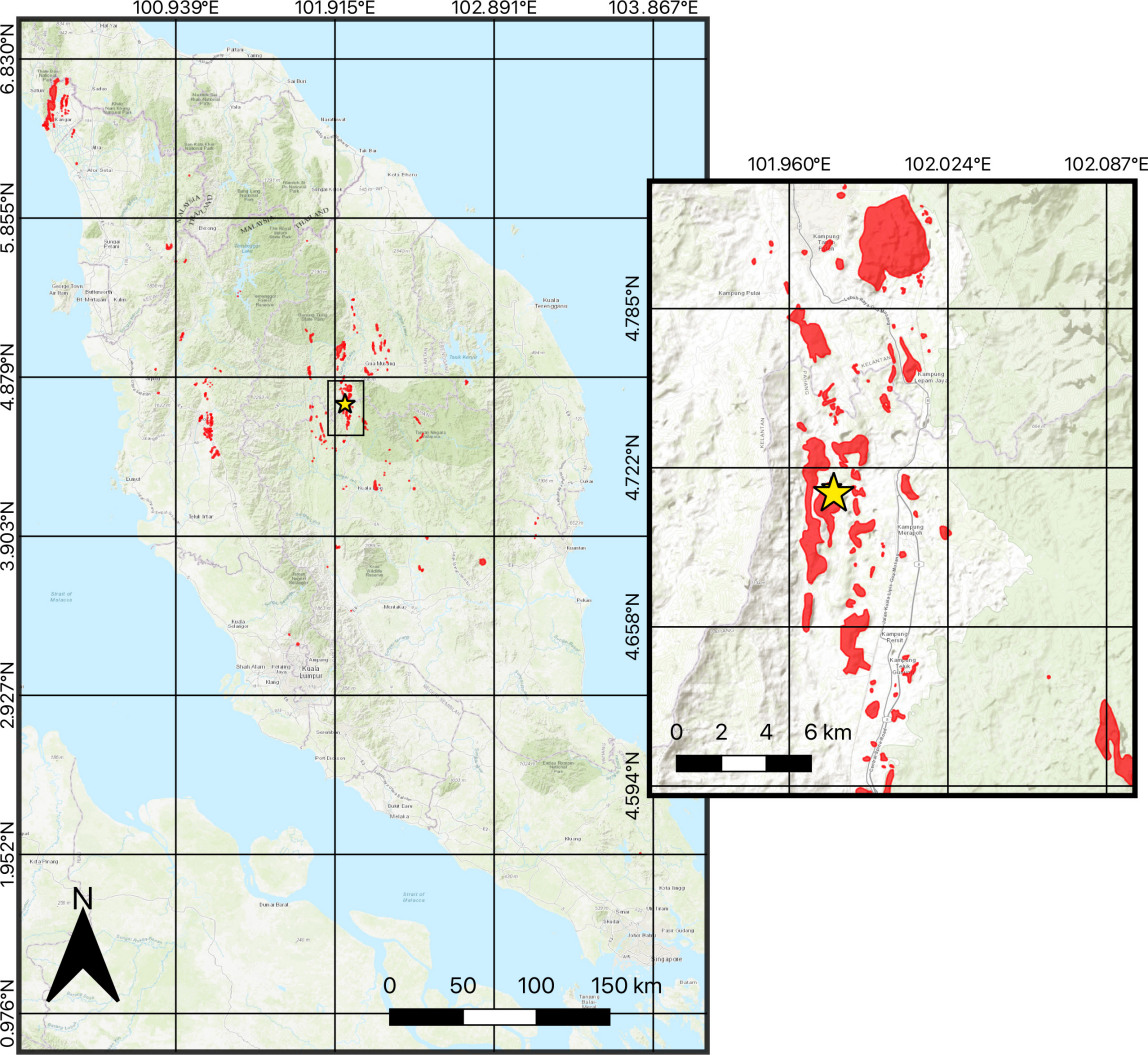
Map of karst areas (in red) in Peninsular Malaysia. Inset map highlighting karst areas around Merapoh town, Pahang state and further north near Gua Musang town, Kelantan state; yellow star indicates location of Gua Hari Malaysia, type locality of *Merapohra karsticola* gen. nov. et sp. nov. (karst data obtained from Liew et al. 2016).

Of the multitude of natural habitats in Peninsular Malaysia, limestone karst areas make up a small percentage of the land area, estimated to be less than 1% of the total land area (Fig. 1; Liew et al. 2016; Quah et al. 2021). Despite taking up a relatively insignificant proportion of the land area in Peninsular Malaysia, karst systems host a disproportionately high diversity of flora and fauna, many of which are endemic (Kiew 1991; Clements et al. 2006, 2008; Grismer et al. 2013, 2014, 2016; Kiew et al. 2017; Quah et al. 2021). Karst landforms in Peninsular Malaysia mostly occur as sheer-sided massif outcrops, usually with caves systems, sinkholes; and their isolation from other stand-alone towers or massifs by non-calcareous landscapes have long been suggested to be a barrier to gene flow for fauna and flora found on separate karsts landforms, resulting in elevated levels of endemism via allopatric and/or parapatric speciation (see van Benthem-Jutting 1958; Tweedie 1961; Schilthuizen et al. 2002). For Peninsular Malaysia, there are more than 1200 species of plants that are associated with karsts, of which, 21% are endemic to the region, and 11% are strictly confined to their respective karsts systems (Chin 1977); while Quah et al. (2021) reported over 63 species of herpetofauna associated with karst systems in Peninsular Malaysia, of which, over a quarter are endemic to both Peninsular Malaysia and their associated karst habitats. Within Peninsular Malaysia, only one species of primary freshwater crab has been described from karst systems/caves, viz., *Phricotelphusa hymieri* Ng & Lee, 2012 (family Gecarcinucidae), from Loposang Cave, Wang Mu Forest Reserve, Perlis state. There is another cavernicolous species endemic to Peninsular Malaysia, viz., *Johora gua* Yeo, 2001 (family Potamidae), which was described from a granitic cave, Gua (cave) Tengkuk Ayer, Gunung (mount) Kajang, on the island of Pulau Tioman, Pahang state. Ng (1988: 2) did note “the presence of an unknown white cave crab from Peninsular Malaysia, but its actual identity remains uncertain since no specimens have been collected so far.” (see also Ng and Yang 1985). The identity of this crab, ostensibly from the Batu Cave systems, however, has never been found.

Recently, specimens of an unusual semi-terrestrial crab were collected from Gua Hari Malaysia, a karstic cave near Merapoh town in Pahang, a state in eastern Peninsular Malaysia. The specimens collected key out to the family Potamidae Ortmann, 1896, sporting a three-article mandibular palp, with the terminal article being simple in morphology (Bott 1970; Ng 1988; Cumberlidge et al. 2024). The specimens, however, cannot be satisfactorily placed in any known genus. A new genus is here-in established for the new species.

## Material and methods

The taxonomic treatment essentially follows Ng (1988), with updates to the terminology following Guinot et al. (2013), Davie et al. (2015), and Cumberlidge et al. (2024). Measurements provided in millimetres are of the maximum carapace width and length, respectively. The specimens examined are deposited in the Zoological Reference Collection (ZRC), Lee Kong Chian Natural History Museum, National University of Singapore (LKCNHM). The following abbreviations and vernacular of some commonly used geographical terms used:

**Amphoe** = district (in Thai);

**Ao** = bay (in Thai);

**asl** = above sea level;

**Ban** = village (in Thai and Lao);

**coll.** = collected by;

**CL** = carapace length;

**CW** = carapace width;

**G1** = male first gonopod;

**G2** = male second gonopod;

**Gua** = cave (in Bahasa Malaysia);

**Gunung** = mount (in Bahasa Malaysia);

**Kampung** = village (in Bahasa Malaysia);

**Khao** = mount (in Thai);

**Mxp3** = third maxilliped;

**Pulau** = island (in Bahasa Malaysia);

**s3/4** = suture between male thoracic sternites 3 and 4;

**Sungei** = river (in Bahasa Malaysia);

**Tambon** = subdistrict (in Thai);

**Tham** = cave (in Thai and Lao);

Specimens were examined with a Leica M80 and M205c stereomicroscope. Camera lucida illustrations were made using the drawing tube mounted on the stereomicroscopes. Monochrome photographs were taken using a Nikon Z 8 mirrorless interchangeable lens camera with NIKKOR Z MC 105 mm F/2.8 VR S lens. Figures were edited and assembled using Adobe Photoshop Lightroom, Adobe Photoshop, maps assembled using QGIS (QGIS.org 2025).

## Taxonomy

### Family Potamidae Ortmann, 1896

**Subfamily Potamiscinae Bott, 1970**

#### Genus Merapohra gen. nov.

Taxon classification

Animalia


BrachyuraPotamidae

B39BDD45-5FC2-5F01-AA2A-9B531C885980

https://zoobank.org/A5B24EEC-728D-4F5F-A31D-55196F7D6085

**Figure 2.**
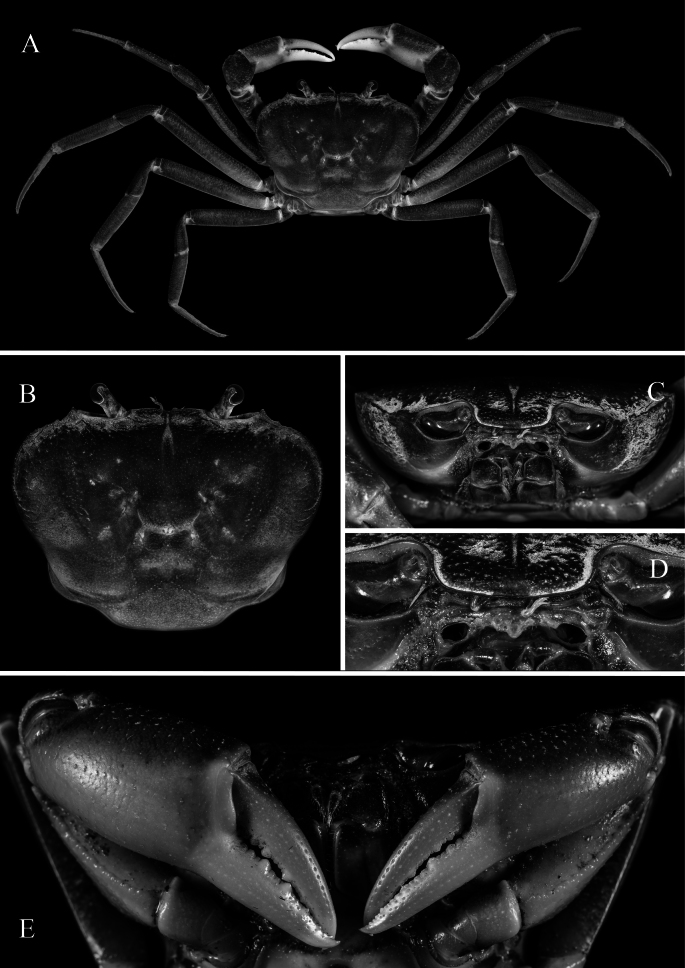
*Merapohra karsticola* gen. nov. et sp. nov. Holotype, male (37.2 × 26.7 mm) (ZRC 2024.0327). **A**. Overall dorsal view; **B**. Dorsal view of carapace; **C**. Frontal view; **D**. Antennae, antennules, and epistome (left side denuded); **E**. Chelipeds.

**Figure 3.**
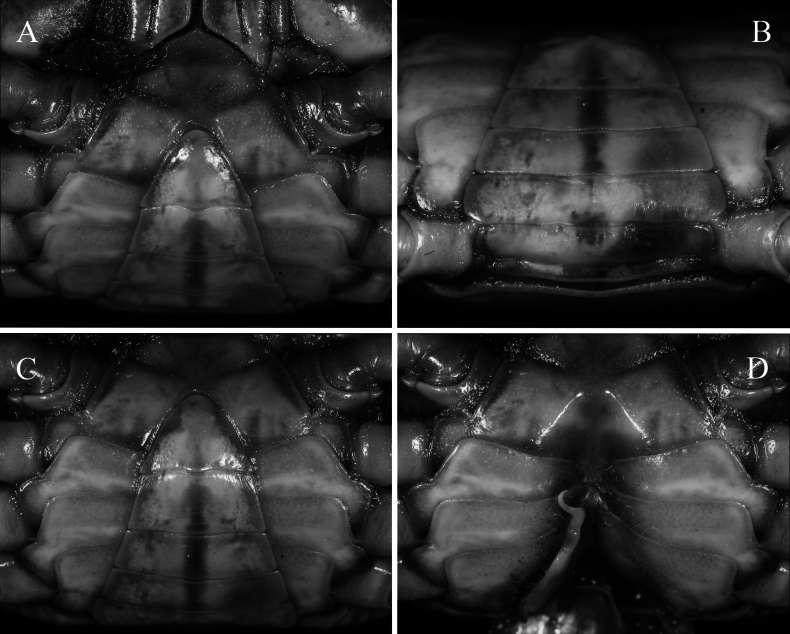
*Merapohra karsticola* gen. nov. et sp. nov. Holotype, male (37.2 × 26.7 mm) (ZRC 2024.0327). **A**. Thoracic sternum, pleonal somite 4–6, and telson (left side denuded); **B**. Pleon somites 1–5; **C**. Posterior thoracic sternum, pleonal somite 3–6, and telson; **D**. Sternopleonal cavity and right G1.

**Figure 4.**
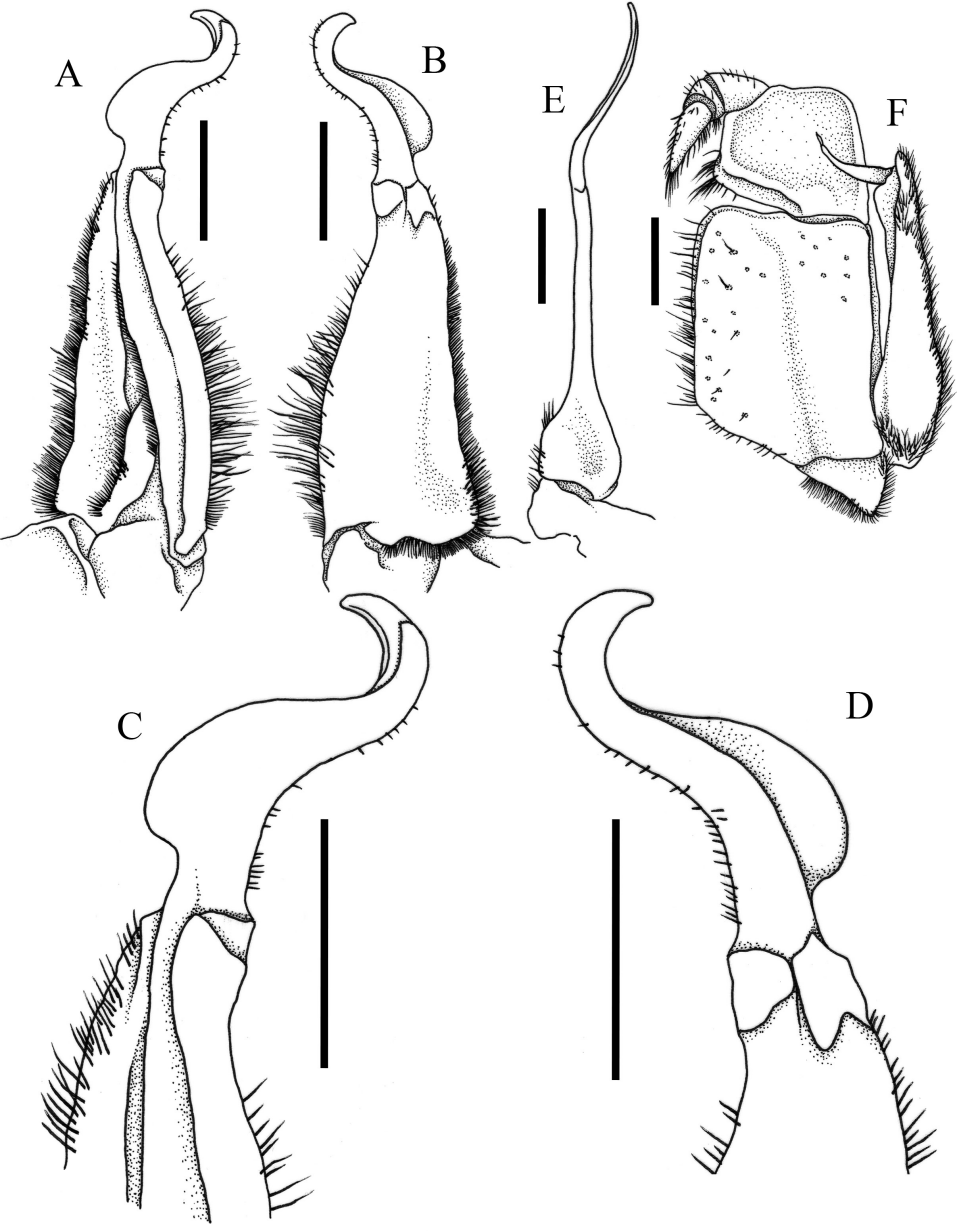
*Merapohra karsticola* gen. nov. et sp. nov. Holotype, male (37.2 × 26.7 mm) (ZRC 2024.0327). **A**. Left G1 (ventral view); **B**. Left G1 (dorsal view); **C**. Distal part of left G1 (ventral view); **D**. Distal part of left G1 (dorsal view); **E**. Left G2; **F**. Left third maxilliped (flagellum set in front of merus, typically positioned behind merus at rest). Scale bar: 2 mm.

**Figure 5.**
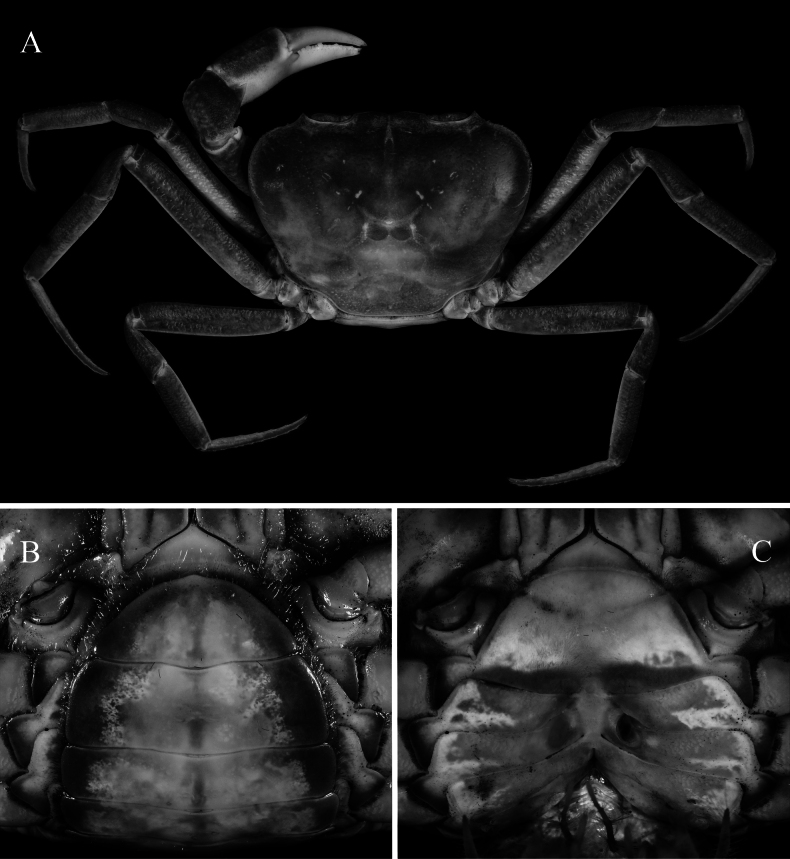
*Merapohra karsticola* gen. nov. et sp. nov. Paratype, female (36.8 × 26.5 mm) (ZRC 2024.0328). **A**. Overall dorsal view; **B**. Pleonal somites 4–6 and telson; **C**. Sternopleonal cavity and vulvae.

**Figure 6.**
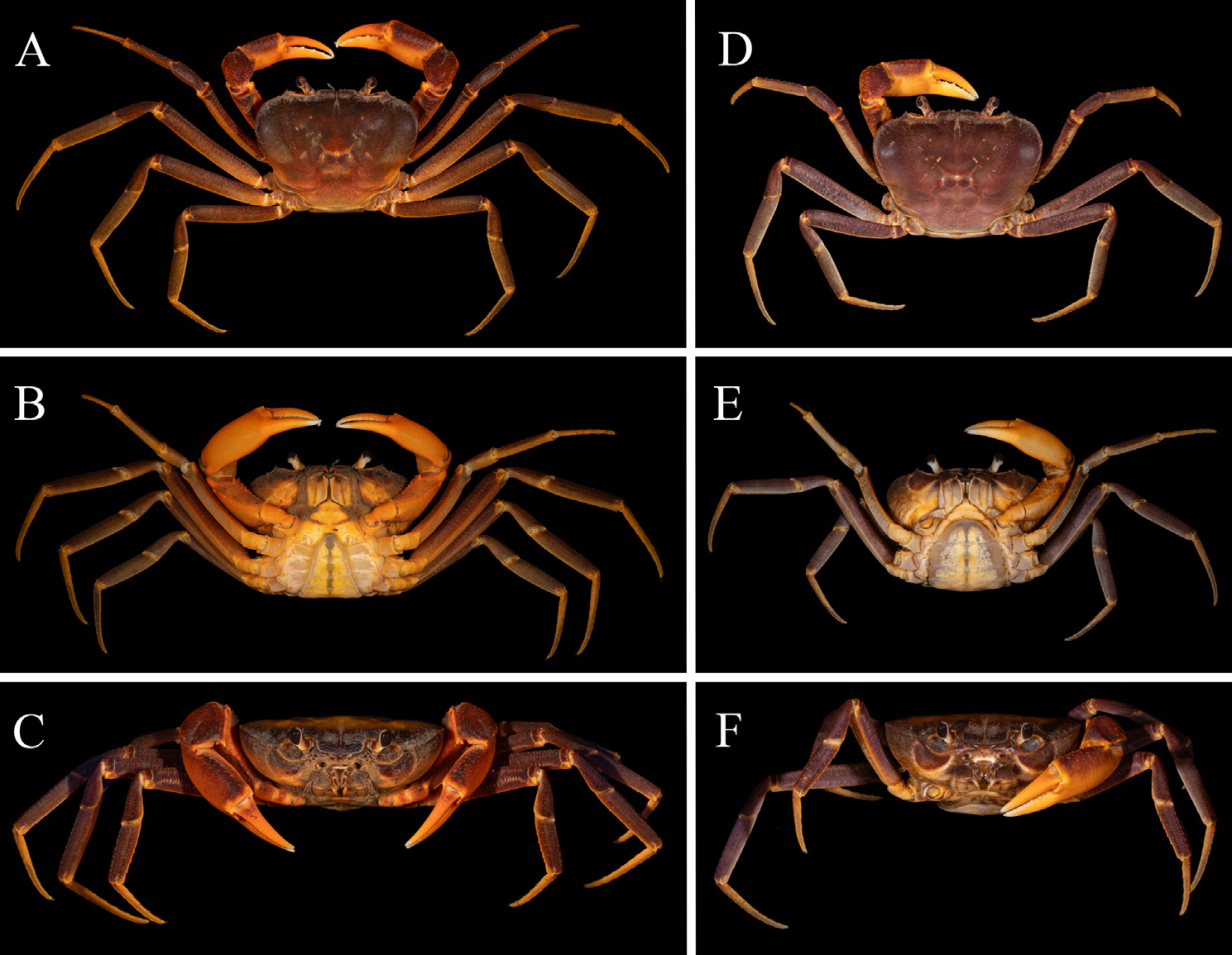
*Merapohra karsticola* gen. nov. et sp. nov. Colour in life (freshly preserved). **A–C**. Holotype, male (37.2 × 26.7 mm) (ZRC 2024.0327); **D–F**. Paratype, female (36.8 × 26.5 mm) (ZRC 2024.0328). **A, D**. Overall dorsal view; **B, E**. Ventral view; **C, F**. Frontal view.

**Figure 7.**
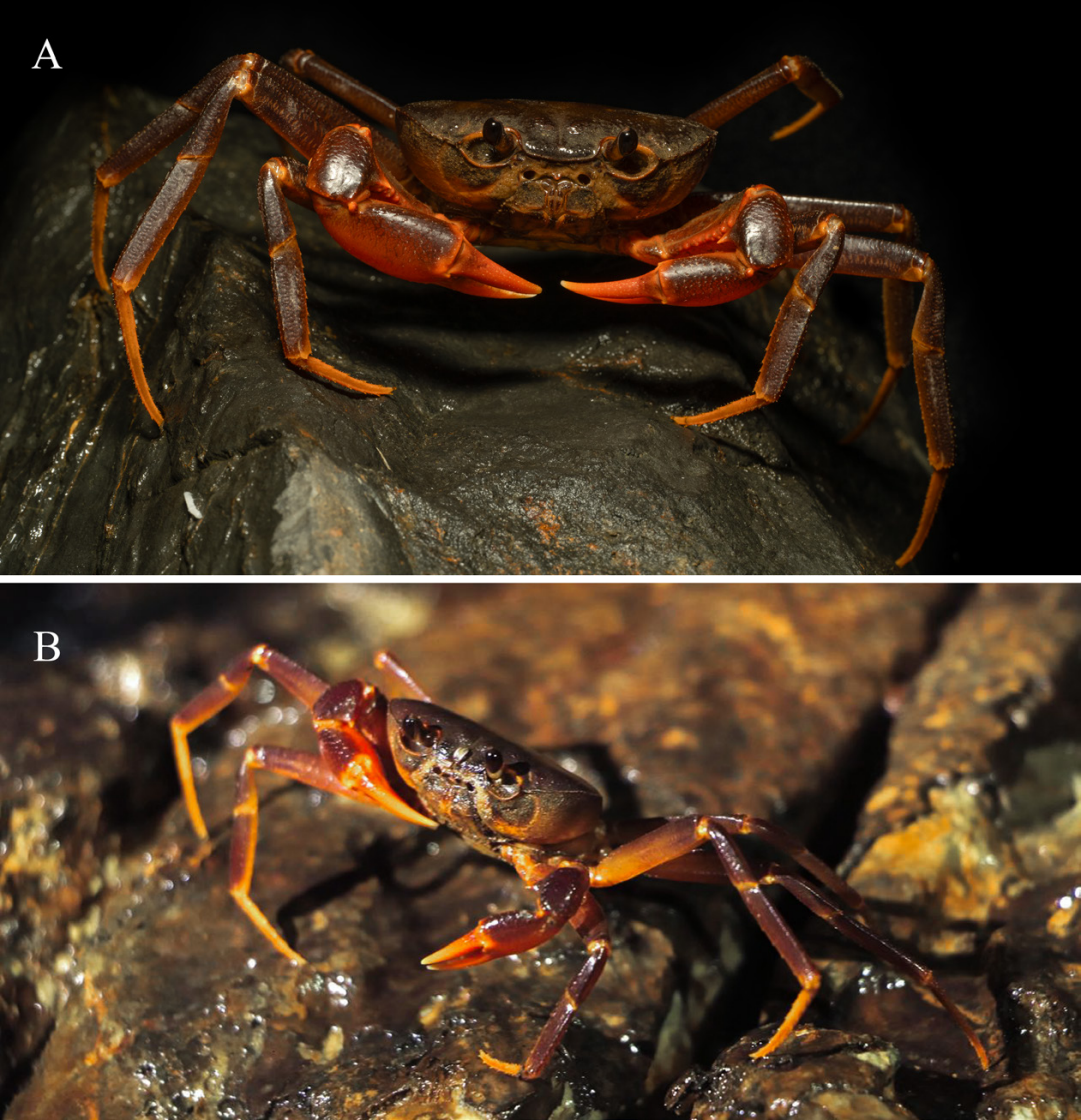
*Merapohra karsticola* gen. nov. et sp. nov., in life. **A**. Holotype, male (37.2 × 26.7 mm) (ZRC 2024.0327); **B**. Paratype, female (30.0 × 21.4 mm) (ZRC 2024.0328). (Photo credit: Mr. Ang Yu Pin for the paratype).

Figs 2, 3, 4, 5, 6, 7

##### Type species.

*Merapohra karsticola* sp. nov. by monotypy.

##### Comparative material.

***Erebusa calobates* Yeo & Ng, 1999. *Holotype***. Laos • male; 20.8 × 17.8 mm; Khammouan Province, Tham Tê, near Ban Na; 11 Feb. 1998; L. Deharveng & A. Bedos leg.; ZRC 1998.1073. ***Paratypes***. Laos • 2 males; 20.0 × 17.4 mm, 19.9 × 17.5 mm; 1 juvenile; same data as holotype; ZRC 1998.1074–1075 • 1 ovigerous female; 23.0 × 19.3 mm; Khammouan Province, Tham Houai Say, near Ban Khen; 26 Feb. 1998; F. Brehier leg.; ZRC 1998.1076.

***Johora grallator* Ng, 1988. *Holotype***. Peninsular Malaysia • female; 33.1 × 23.5 mm; Pahang, Pulau Tioman, Gunung Kajang, 792 m asl, Ulu Lalang, in insect trap baited with coconut, ca 2°51'23"N, 104°09'37"E; 18 Jun. 1962; J.A. Bullock leg.; ZRC 1989.2691. ***Non-type specimens***. Peninsular Malaysia • 2 juvenile males; 11.2 × 8.8 mm, 11.9 × 9.0 mm; Pahang, Pulau Tioman, Gunung Kajang, under rock in dry cave; 27 Jun. 1996; T.H.T. Tan et al. leg.; ZRC 1996.1730 • 1 male; 32.7 × 23.1 mm; Pahang, Pulau Tioman, Gunung Kajang, ca 700 m asl, on trail; 8 Jul. 2024; J. Bartels leg.; ZRC 2024.0159.

***Johora gua* Yeo, 2001. *Holotype***. Peninsular Malaysia • male; 11.4 × 8.5 mm; Pulau Tioman, Gua Tengkuk Ayer, route to Gunung Kajang from Juara, ca 900 m asl; 1 Sep. 2000; H.H. Tan & T.M. Leong leg.; ZRC 2000.2236.

***Johora punicea* (Ng, 1985). *Holotype***. Peninsular Malaysia • male; 19.7 × 15.5 mm; Pahang, Pulau Tioman, under rocks, on slope adjacent to Sungei Besar, ca 300 m asl; 20 Jun. 1984; P.K.L. Ng leg.; ZRC 1984.6803. ***Paratypes***. Peninsular Malaysia • 1 male; 21.6 × 16.0 mm; 2 females; same data as holotype; ZRC 1984.6804–6806. ***Non-type specimens***. Peninsular Malaysia • 1 female; 19.9 × 15.6 mm; Pahang, Pulau Tioman, Paya; Jul. 2018; Honours Class leg.; ZRC 2022.0102 • 1 ovigerous female; Pahang, Kampung Paya; 19 Sep. 1995; P.K.L. Ng leg.; ZRC 2018.1130 • 1 male; Pahang, Pulau Tioman, Kampung Paya; Jul. 2005; H.H. Tan leg.; ZRC 2018.1113.

***Demanietta sunthorni* Lheknim, Leelawathanagoon & Ng, 2023. *Holotype***. Thailand • male; 36.6 × 27.7 mm; Ao Dan, south of Adang Island, unnamed creek, 6°30.732'N, 99°18.226'E, 35 m asl; 20 Oct. 2008; V. Lheknim & P. Leelawathanagoon leg.; ZRC 2022.0826.

***Phaibulamon stilipes* Ng, 1992. *Holotype***. Thailand • male; 28.5 × 22.7 mm; Kanchanaburi Province, Kwai Valley, Tham Nam Pah Khoan cave, from “K2” resurgence; 18 Aug. 1990; French Expedition Kwai 90 leg.; ZRC 1992.8325.

***Terrapotamon abbotti* (Rathbun, 1898). *Non-type specimens***. Thailand • 3 males; 39.2 × 32.3 mm, 29.5 × 24.3 mm, 25.7 × 20.8 mm; 1 female; 33.4 × 27.8 mm; Surat Thani Province, Bon Baeng Bap, Ratthanikhom, Amphoe Khiri; 16–19 Jul. 1983; T. Sisuchat & D. Kuntisit leg.; ZRC 2012.0683.

***Terrapotamon longitarsus* Lheknim & Ng, 2016. *Holotype***. Thailand • male; 47.4 × 38.6 mm; Satun Province, Muang Satun District, Tambon Khuan Po, found dead on limestone bedrock at Khao Raya Bung Sa, southernmost of Nakhon Si Thammarat mountain range, 30 m asl; 9 Sep. 1999; V. Lheknim & P. Leelawathanagoon leg.; ZRC 2016.0161.

***Terrapotamon palian* Ng & Naiyanetr, 1998. *Holotype***. Thailand • male; 24.6 × 20.5 mm; Trang Province, Palian District, Ton Tok Waterfall; 21 May 1976; P. Naiyanetr leg.; ZRC 2012.0685.

***Thampramon tonvuthi* Ng & Vidthayanon, 2013. *Non-type specimens***. Thailand • 1 male; 33.9 × 27.8 mm; Baan Chompu southwest of Thung Salaeng Luang National Park, Tham Phra Wangdaeng, cave entrance, 16.8379°N, 100.877°E; 8 Oct. 1997; C. Vidthayanon leg.; ZRC 2012.1126 • 1 female; 42.7 × 36.3 mm; same locality as male, in twilight zone of cave, ca 100 m from entrance, on wet rock wall, 4 m above stream above bat guano; 28 Aug. 2002; D. Smart leg.; ZRC 2012.1127.

***Nemoron nomas* Ng, 1996. *Holotype***. Vietnam • male; 23.7 × 20.3 mm; Quang Binh Province, Hang Toi, Phong Nha, station Viet 062, 35 m asl, in cave, about 300 m inside; 11 Jan. 1995; L. Deharveng, A. Bedos & Levet leg.; ZRC 1996.0094.

***Tiwaripotamon edostilus* Ng & Yeo, 2001. *Holotype***. Vietnam • male; 36.4 × 28.3 mm; Ha Long Bay, Cat Ba Island, cave at Gia Luang; 28 Sep. 1998; L. Deharveng leg.; ZRC 2000.0096. ***Paratype***. Vietnam • male; 40.5 × 31.1 mm; same data as holotype; ZRC 2000.0097.

##### Diagnosis.

Carapace (Figs 2A, 2B, 5A) transversely subovate, slightly wider than long, CW/CL ratio 1.30–1.40; dorsal surface gently convex, smooth, glabrous; branchial regions not prominently expanded laterally, not inflated in frontal view, lateral branchial regions not discernible in dorsal view; orbits oblique in frontal view; epigastric and postorbital cristae not clearly discernible, appearing as smooth margin with small granulations, epibranchial crista slightly anterior of postorbital crista, postorbital crista with lateral part breaking up into low striae and granules approaching but not joining anterolateral margin; postorbital, postfrontal and epibranchial regions with indistinct low granules, rugae and/or punctae (Fig. 2D); epibranchial tooth poorly defined, weakly separated from external orbital tooth by shallow cleft; external orbital tooth well produced, narrowly triangular with concave lateral margins, outer margin ~ 1.77 × length of inner margin, just before suborbital margins; margins of posterior epistomal margin with low median lobe, lateral parts gently sloping downwards, not parallel with frontal margin (Fig. 2E). Third maxilliped with subquadrate merus, ischium rectangular, length to width ratio 1.40, exopod relatively slender, outer margin straight, flagellum short, approx. half-length of merus (Fig. 4F). Ambulatory legs and dactylus (Fig. 2A) slender, elongate, margins and surfaces smooth. Male thoracic sternites 1 and 2 fused, relatively narrow, width-to-length ratio 2.87, sternites 3 and 4 fused, relatively narrow, but with shallow, visible grooves demarcating sutures; thoracic sternite 8 completely hidden when male pleon closed (Fig. 3A, B); male pleon (Fig. 3B, C) broadly triangular; male telson broadly triangular, lateral margins almost straight. Male sternopleonal cavity (Fig. 3D) reaching imaginary line joining median point of cheliped coxae; press-button tubercle of male pleonal locking mechanism distinct, on posterior third of sternite 5. G1 subterminal article (Fig. 4A–D) relatively stout, straight; terminal article long, 0.6 × length of subterminal article, strongly curving outwards anteriorly before recurving posteriorly, gently tapering throughout to broad tip, with long, medial dorsal flap. Vulva (Fig. 5C) conspicuously large, directed mesial-ventrally, occupying almost length of sternite 6, impinging upwards onto sternite 5, sternite 5 forming ledge-like overhang over sternite 6 and vulvae; intervulvar distance, measured between mesial margins of both vulvae, 0.42 × vulvar distance, measured between lateral margins of both vulvae.

##### Etymology.

The new genus is named after the town of Merapoh in Lipis district, Pahang state, Peninsular Malaysia; an area known for numerous limestone hills and caves. Gender feminine.

##### Remarks.

The external morphology of *Merapohra* gen. nov., is similar to 25 karst dwelling potamid crabs found in Southeast Asia: *Cerberusa tipula* Holthuis, 1979, *C. caeca* Holthuis, 1979 (both from Sarawak, Borneo, Malaysia); *Erebusa calobates* Yeo & Ng, 1999 (Khammouan Province, central Laos); *Kanpotamon duangkhaei* Ng & Naiyanetr, 1993 (Kanchanaburi Province, western Thailand); *Nemoron nomas* Ng, 1996 (Quang Binh Province, central Vietnam); *Ngan mayla* Guinot & Rodríguez Moreno, 2024 (East Kalimantan, Borneo, Indonesia); *Phaibulamon stilipes* Ng, 1992 (Kanchanaburi Province, western Thailand); *Schubartamon serenum* Guinot & Rodríguez Moreno, 2024 (Vientiane Province, central Laos); *Terrapotamon longitarsus* Lheknim & Ng, 2016, *Te. thungwa* Promdam, Yeesin & Ng, 2017 (both from Nakhon Si Thammarat, Satun, Surat Thani, Trang Provinces, southern Thailand); *Thampramon tonvuthi* Ng & Vidthayanon, 2013 (Phetchabun Province, central Thailand: one species); and 14 species of *Tiwaripotamon* Bott, 1970 (from northern Vietnam and Guangxi Province, southern China); especially with regards to their dolichopodous form which are likely adaptations for life in karstic habitats. Of these taxa, *Erebusa* Yeo & Ng, 1999, *Kanpotamon* Ng & Naiyanetr, 1993, *Ngan* Guinot & Rodríguez Moreno, 2024, *Phaibulamon* Ng, 1992, *Schubartamon* Guinot & Rodríguez Moreno, 2024, and *Thampramon* Ng & Vidthayanon, 2013, are monotypic genera, while *Cerberusa* Holthuis, 1979, only has two known species. *Terrapotamon* Ng, 1986, has five known species but only the two listed here have long ambulatory legs, the other possessing short legs and are not found near caves (see Ng 1988; Leelawathanagoon et al. 2010; Promdam et al. 2017).

*Merapohra* can be immediately distinguished from *Cerberusa*, *Ngan*, *Nemoron* Ng, 1996, and *Phaibulamon* by the presence of a distinct albeit short flagellum on the exopod of the third maxilliped, a character absent in the latter four genera (Fig. 4F, Table 1; cf. Holthuis 1979; Ng 1992c: fig. 2A; Ng 1996: fig. 2A; Guinot and Rodríguez Moreno 2024a). Among the remaining taxa that possess a flagellum on the third maxilliped exopod, *Erebusa* and *Schubartamon* differ markedly from *Merapohra* in possessing very short eyestalks; in *Merapohra*, the longer eye stalks are distinctly longer, sporting well developed and corneas pigmented, suggesting a more epigean or trogophilic lifestyle (Fig. 2B, C, Table 1; cf. Yeo and Ng 1999: fig. 1E; Guinot and Rodríguez Moreno 2024b: figs 3B, 7B, 8).

**Table 1.** Morphological differences between *Merapohra*, new genus, and other similar karst dwelling potamid genera in Southeast Asia.

Morphological character	*Merapohra* gen. nov.	*Cerberusa* Holthuis, 1979	*Erebusa* Yeo & Ng, 1999	*Kanpotamon* Ng & Naiyanetr, 1993	*Nemoron* Ng, 1996	*Ngan* Guinot & Rodríguez Moreno, 2024	*Phaibulamon* Ng, 1992	*Schubartamon* Guinot & Rodríguez Moreno, 2024	*Terrapotamon* Ng, 1986	*Thampramon* Ng & Vidthanayon, 2013	*Tiwaripotamon* Bott, 1970
Carapace	sub-ovate	sub-ovate	sub-ovate	sub-ovate	sub-quadrate	sub-quadrate	sub-quadrate	sub-ovate	sub-ovate	sub-ovate	sub-ovate
Carapace height	low	low	high	low	high	low	low	low	high	low	low
Carapace dorsal surface	mostly smooth, branchial region gently granulose	smooth	mostly smooth, posterolateral margin with granules and striae	smooth	granulose and rugose around anterolateral, gastric, frontal, and postorbital regions	smooth	strongly granulose and rugose throughout	finely granulated throughout	mostly smooth, branchial region gently rugose/granulose	mostly smooth, branchial region gently rugose	smooth
Pterygostomial and sub-hepactic regions	completely smooth	weakly rugose or rugose	smooth to finely granulated	smooth	rugose and granulose	smooth	strongly granulose and rugose	finely granulated	granulose	gently rugose	smooth
Postorbital and epigastric cristae	not well-defined, very weakly granulose	not well-defined, smooth	not well- defined, rounded	well-defined, rugose	well-defined, strongly rugose	defined, as ridges	defined, strongly granulated	not well- defined, rounded	well- defined, rounded with granules	well- defined, sharp	not well-defined, smooth
Anterolateral margin	sub-cristate, very weakly serrated	sub-cristate, smooth	sub-cristate, weakly serrated	cristate, serrated	cristate, serrated	sub-cristate, smooth	cristate, serrated	sub-cristate, weakly serrated	cristate, strongly serrated	cristate, strongly serrated	gently serrated to sub-cristate
Eye stalk	long	short	short	long	long	short	long	short	long	long	long
Mxp3 exopod	exceeding ischium	exceeding ischium	exceeding ischium	exceeding ischium	not exceeding ischium	exceeding ischium	not exceeding ischium	exceeding ischium	exceeding length of ischium	exceeding length of ischium	exceeding length of ischium
Mxp3 flagellum	long	absent	long	long	absent	absent	absent	long	long	long	long
Antennular fossa	narrow	broad	broad	broad	broad	broad	broad	broad	narrow	narrow	narrow
G1 terminal article	broad, strongly recurved upwards distally, with dorsal flap	broad, curving downwards, no dorsal flap	very slender, curving downwards, very small dorsal flap	very broad, gently curving downwards, with bulbous structure on proximal part	broad, gently curving downwards, tall, large hump-like dorsal flap	unknown*	broad, bent outwards 90°, tip gently recurved, no dorsal flap	unknown*	broad, straight, without dorsal flap	broad, curving downwards, with tall, hump-like, notched dorsal flap	broad, gently recurved distally, with very low or no dorsal flap

* Original description based on female only.

*Kanpotamon duangkhaei*, *Terrapotamon longitarsus*, *Te. thungwa*, *Thampramon tonvuthi*, and members of *Tiwaripotamon* all have short to very short flagella on the exopods of their third maxillipeds and large pigmented eyes on longer eye stalks, which are more similar to *Merapohra*. The two *Terrapotamon* species are the most dissimilar, distinguishable by its higher, more subquadrate carapace, that is more strongly covered in rugosities and granules particularly around branchial, sub-branchial, pterygostomial regions, and anterolateral margins (cf. Lheknim and Ng 2016: fig. 1A; Promdam et al. 2017: fig. 2A); and a more strongly defined epigastric, postorbital cristae, anterolateral margin, and epibranchial tooth (cf. Lheknim and Ng 2016: fig. 1A, B, C; Promdam et al. 2017: fig. 2A, B). In *Merapohra*, the carapace is relatively flatter, more transversely sub-ovate, that is very weakly rugose or completely smooth on both dorsal and ventral surfaces (Fig. 2B, C), with a much more weakly defined epigastric, postorbital cristae, anterolateral margin, and epibranchial tooth (Fig. 2B) (Table 1).

In comparison to the species of *Kanpotamon*, *Thampramon*, and *Tiwaripotamon*, the new genus *Merapohra* can be differentiated by the following morphological differences: 1) carapace is transversely sub-ovate, relatively smooth, except for few gentle rugosities and granules around branchial regions (Fig. 2B) (vs carapace less transverse, more strongly rugose around branchial regions in *Thampramon*, while in *Kanpotamon* and *Tiwaripotamon*, it is completely smooth, cf. Ng and Naiyanetr 1993: fig. 21; Ng and Yeo 2001: figs 1A, 3A, 4A; Ng and Vidthayanon 2013: fig. 2; Shih and Do 2014: figs 4A, 6A; Do et al. 2016: figs 3A, 4A, 5A; Do et al. 2017: figs 2A, 5A; Ng and Ngo 2022: fig. 2A; Ng 2024: fig. 1A; Dang and Do 2025: fig. 2A); 2) anterolateral margin sub-cristate, very weakly serrated (Fig. 2B) (vs anterolateral margins strongly cristate, appearing raised and serrated in *Kanpotamon* and *Thampramon*, while in *Tiwaripotamon*, the anterolateral margins can appear smooth to serrated, cf. Ng and Naiyanetr 1993: fig. 21; Ng and Yeo 2001: figs 1A, 3A, 4A; Ng and Vidthayanon 2013: fig. 2; Shih and Do 2014: figs 4A, 6A; Do et al. 2016: figs 3A, 4A, 5A; Do et al. 2017: figs 2A, 5A; Ng and Ngo 2022: fig. 2A; Ng 2024: fig. 1A; Dang and Do 2025: fig. 2A); 3) epibranchial tooth less well-defined, appearing as a nodule, confluent with external orbital tooth (Fig. 2B) (vs epibranchial tooth large, triangular, clearly separated from external orbital tooth by deep cleft in *Kanpotamon* and *Thampramon*, that in *Tiwaripotamon*, varies in size, from broad angle to triangular, still clearly separated from external orbital tooth, cf. Ng and Naiyanetr 1993: fig. 21; Ng and Yeo 2001: figs 1A, 3A, 4A; Ng and Vidthayanon 2013: fig. 2; Shih and Do 2014: figs 4A, 6A; Do et al. 2016: figs 3A, 4A, 5A; Do et al. 2017: figs 2A, 5A; Ng and Ngo 2022: fig. 2A; Ng 2024: fig. 1A; Dang and Do 2025: fig. 2A); 4) both epigastric and postorbital cristae poorly developed, appearing smooth except for few granules at lateral margins (Fig. 2B) (vs epigastric and postorbital cristae well-developed, appearing sharp in *Kanpotamon* and *Thampramon*, that in *Tiwaripotamon* is even more poorly defined, completely smooth, cf. Ng and Naiyanetr 1993: fig. 21; Ng and Yeo 2001: figs 1B, 3B, 4B; Ng and Vidthayanon 2013: fig. 3; Shih and Do 2014: figs 4B, 7B; Do et al. 2016: figs 3B, 4B, 5B; Do et al. 2017: figs 2B, 5B; Ng and Ngo 2022: fig. 3B; Ng 2024: fig. 2F, G; Dang and Do 2025: fig. 2A); and 5) epistomal medial lobe, well produced, narrowly triangular (Fig. 2D) (vs epistomal median lobe lower, appearing as broad triangle in *Thampramon* and *Tiwaripotamon*, cf. Ng and Yeo 2001: figs 1A, 3A, 4A; Ng and Vidthayanon 2013: fig. 2; Shih and Do 2014: figs 4A, 6A; Do et al. 2016: figs 3A, 4A, 5A; Do et al. 2017: figs 2A, 5A; Ng and Ngo 2022: fig. 2A; Ng 2024: fig. 1A; Dang and Do 2025: fig. 2B).

Perhaps the most significant feature of *Merapohra* gen. nov., is in its unique G1 morphology, where the terminal article is relatively long, about 0.6 × length of subterminal article, strongly curving outwards proximally, and nearly recurved 180° distally, with a large semi-circular dorsal flap (Fig. 4B, D). No other potamid crab possesses a G1 with such a strongly recurved distal terminal article. This contrasts with other long-legged karst dwelling species in Southeast Asia, which typically possess a terminal article that is proportionally shorter, about 0.2–0.4 × length of subterminal article, which in *Kanpotamon*, *Terrapotamon*, and *Thampramon*, is not recurved, while in *Tiwaripotamon*, it can range from curving downwards to being gently recurved, either without a dorsal flap, or if present, is always small and low, cf. Ng and Naiyanetr 1993: fig. 55; Ng and Yeo 2001: figs 2D–H, 5D–K; Leelawathanagoon et al. 2010: fig. 1F, H; Ng and Vidthayanon 2013: fig. 7C, D; Shih and Do 2014: fig. 3A–H; Do et al. 2016: fig. 2A–F; Lheknim and Ng 2016: fig. 5; Do et al. 2017: fig. 4A–C, 7A–D; Promdam et al. 2017: fig. 4; Ng and Ngo 2022: fig. 5A–D; Ng 2024: fig. 5A–D, F–H; Dang and Do 2025: fig. 5B, C, E–G).

The only other cavernicolous potamid crab species from Peninsular Malaysia is *Johora gua* Yeo, 2001, described from Gua Tengkuk Ayer, near the summit of Gunung Kajang, Pulau Tioman. *Johora gua* is an unusual species, for the genus, featuring troglomorphic features, including distinctly elongated legs and reduced cornea size (Yeo 2001), that is unlike most species of *Johora*, which typically have stouter ambulatory legs and regular sized cornea (Ng 1988, 2020). Nevertheless, *Merapohra* differs from *Johora gua* and all other species of *Johora* by its very poorly developed, rounded epigastric and postorbital cristae; nearly indiscernible epibranchial tooth; and a G1 terminal article that is strongly recurved with a pronounced dorsal flap (Fig. 4B, D). This contrasts with all *Johora* species that are characterised by their well-defined epigastric cristae, postorbital cristae, and epibranchial tooth; and a G1 terminal article that ranges from being straight to curving downwards, never recurved, typically without dorsal flap, if ever present, is very low (cf. Ng 1988: figs 13–23; Yeo 2001: figs 1A, 2C–F; Leelawathanagoon et al. 2005: fig. 2; Ng 2020: figs 2, 6–12).

There are some potamids that have been collected in karstic caves in Southeast Asia that have relatively short ambulatory legs, but these species do not have any characters that suggest they are troglophiles or troglobites. For example, *Isolapotamon bauense* Ng, 1987 (Sarawak, Malaysia), *Shanphusa ywarngan* Ng & Whitten, 2017, and *Demanietta burmanica* Ng, 2018 (both Myanmar), were collected from caves but none have cave adapted features (Ng 1987, 2018b; Ng and Tan 1998; Ng and Whitten 2017) and are now regarded as troglophiles at most. The holotype of *Xestomon tacu* Ng, 2021, was collected from a cave in southern Vietnam; and the species is overall pale orange in colour, but the corneas are fully pigmented, and specimens have also been collected in several epigeal habitats (Ng 2021).

#### Merapohra karsticola sp. nov.

Taxon classification

Animalia


BrachyuraPotamidae

7734DAEF-3637-52D4-A857-87DF8A64B60D

https://zoobank.org/A16EBFCD-BA6A-48B2-8775-AD931997C3CB

Figs 2, 3, 4, 5, 6, 7

##### Type material.

***Holotype***. Peninsular Malaysia • male; 37.2 × 26.7 mm; Pahang State, Merapoh, Gua Hari Malaysia; 19 Apr. 2025; Q. Ooi et al. leg.; ZRC 2024.0327. ***Paratypes***. Peninsular Malaysia • 3 females; 36.8 × 26.5 mm, 30.0 × 21.4 mm, 20.0 × 15.4 mm; same data for holotype; ZRC 2024.0328.

##### Diagnosis.

As for new genus.

##### Description of male holotype.

Carapace subquadrate, anteriorly wider than long, relatively low; dorsal surfaces smooth, except for few granules around branchial regions, regions poorly demarcated, cervical grooves barely visible; H-shaped gastro-cardiac depression shallow but discernible (Fig. 2A, B); dorsal surface (including branchial regions) gently convex, gently inflated (Fig. 2C); epigastric cristae very low, marked by two low humps; postorbital cristae barely visible, rounded (Fig. 2B). Frontal margin gently convex, lobes not well marked, appearing almost straight in dorsal view, contiguous with supraorbital margin (Fig. 2B, C). Supraorbital margin gently sinuous, without clefts or fissures (Fig. 2C). External orbital tooth, short, broad, margins distinctly concave, outer margin longer than inner margin, outer margin ~ 1.77 × length of inner margin, just before suborbital margin; barely demarcated from anterolateral margin by minute indentation (Fig. 2B); epibranchial tooth low, present as small tubercle, weakly separated from external orbital tooth by shallow cleft; rest of anterolateral margin convex, subcristate; posterolateral margin gently sinuous, converging towards gently convex posterior carapace margin (Fig. 2B). Orbits ovate, large; eyes nearly filling space; cornea large, pigmented black; peduncle not elongate (Fig. 2B, C). Antennules long, folding laterally into narrow, subrectangular antennular fossa (Fig. 2D); antennae short, shorter than peduncle (Fig. 2D). Epistome longitudinally narrow, lateral parts covered in dense short hairs; posterior margin with low, triangular median lobe, confluent with gently sinuous lateral margins; margins of posterior epistomal margin lateral to median lobe gently sloping downwards, not parallel with frontal margin (Fig. 2D). Suborbital margin concave, smooth, without inner tooth; suborbital region smooth; subhepatic, pterygostomial and sub-branchial regions weakly rugose to smooth (Fig. 2C).

Third maxillipeds relatively short; merus subquadrate, shorter and narrower than ischium, surface slightly pitted, otherwise smooth and generally glabrous; ischium rectangular, length to width ratio 1.4, median sulcus shallow; exopod relatively slender, long, outer margin straight, nearly reaching to half-length of merus, with short flagellum approx. half-length of merus (Figs 2C, 4F).

Chelipeds elongate; right one slightly larger (Fig. 2A, E). Basis-ischium with few granules on ventral margin; ventral margins of merus lined with tubercles, dorsal margin uneven, gently rugose; carpus with broad spine on inner angle, surface gently rugose; outer surface of chela almost smooth, surface of fingers punctate; fingers slightly shorter than palm in major chela, equal in minor chela; fingers gently curved with large cutting teeth evenly spread out (Fig. 2A, E).

Ambulatory legs relatively long, slender, margins and surfaces smooth; second leg longest, fourth leg shortest; dorsal margins sub-cristate, appearing very weakly serrate; carpus with subdorsal ridge; propodus long, with shallow median sulcus; dactylus long, subequal in length to propodus, gently curved (Fig. 2A).

Anterior thoracic sternum relatively narrow; surface of sternites smooth, glabrous except for dense short setae on lateral margins (Fig. 3A, C). Sternites 1 and 2 completely fused forming triangular plate with convex lateral margins, relatively narrow, width-to-length ratio 2.87, separated from sternite 3 by distinctly convex suture (anteriorly); sternites 3 and 4 fused, s3/4 present as shallow lateral groove that is medially interrupted by anterior edge of sternopleonal cavity; s4/5, s5/6 and s6/7 medially interrupted; deep longitudinal groove between sternite 8 extending to median part of sternite 7 (Fig. 3A, D). Somite 8 not visible when pleon closed (Fig. 3B). Sternopleonal cavity reaching to imaginary line connecting median part of coxae of chelipeds; tubercle of male pleonal locking mechanism on posterior third of somite 5 (Fig. 3A, D).

Pleon broadly triangular; somite 1 longitudinally narrow, reaching to edges of coxae of fourth ambulatory legs (Fig. 3B); somite 2 of same width as somite 1; somite 3 widest; somites 4 and 5 trapezoidal; somite 6 trapezoidal, wide; telson broadly triangular, lateral margin almost straight (Fig. 3A–C).

G1 relatively short, stout; subterminal article relatively stout, straight, basal part distinctly broad, before tapering distally, without neck-like distal portion; terminal article elongated, slender, about 0.6 × length of subterminal article, very strongly curving outwards anteriorly before recurving posteriorly, gently tapering along length to broad tip, with relatively long, medial dorsal flap (Fig. 4A–D).

##### Female paratypes.

The female specimens agree with the holotype in all non-sexual characters except for its more slender and more symmetrical chelipeds (Fig. 5A). The thoracic sternum has the same features except that it is proportionately wider (Fig. 5C). The pleon is ovate and covers almost the entire surface of the thoracic sternum when closed (Fig. 5B). Vulva is very large, directed ventrally, occupying almost length of sternite 6, impinging upwards onto sternite 5, sternite 5 forming a ledge-like overhang over sternite 6 and vulvae; intervulvar distance, measured between mesial margins of both vulvae, 0.42 × vulvar distance, measured between lateral margins of both vulvae (Fig. 5C).

##### Etymology.

The specific epithet is derived from a combination of the word karst (limestone) and the Latin suffix -*colo* for tendency to inhabit or dwell in, alluding to the karst dwelling habits of this new species.

##### Live colouration.

In life, the dorsal surfaces of the carapace, ambulatory legs, and the chelipeds are purple in colour. The joints of ambulatory legs, chelipeds, outer surfaces of chelipeds and margins of orbits are bright orange. The ventral surfaces largely pale off-white to light orange in colouration (Figs 6, 7).

##### Remarks.

Differences between morphologically close, karst-dwelling species from the region have been elaborated at length in the remarks section for the new genus (see above).

##### Distribution.

**Figure 8.**
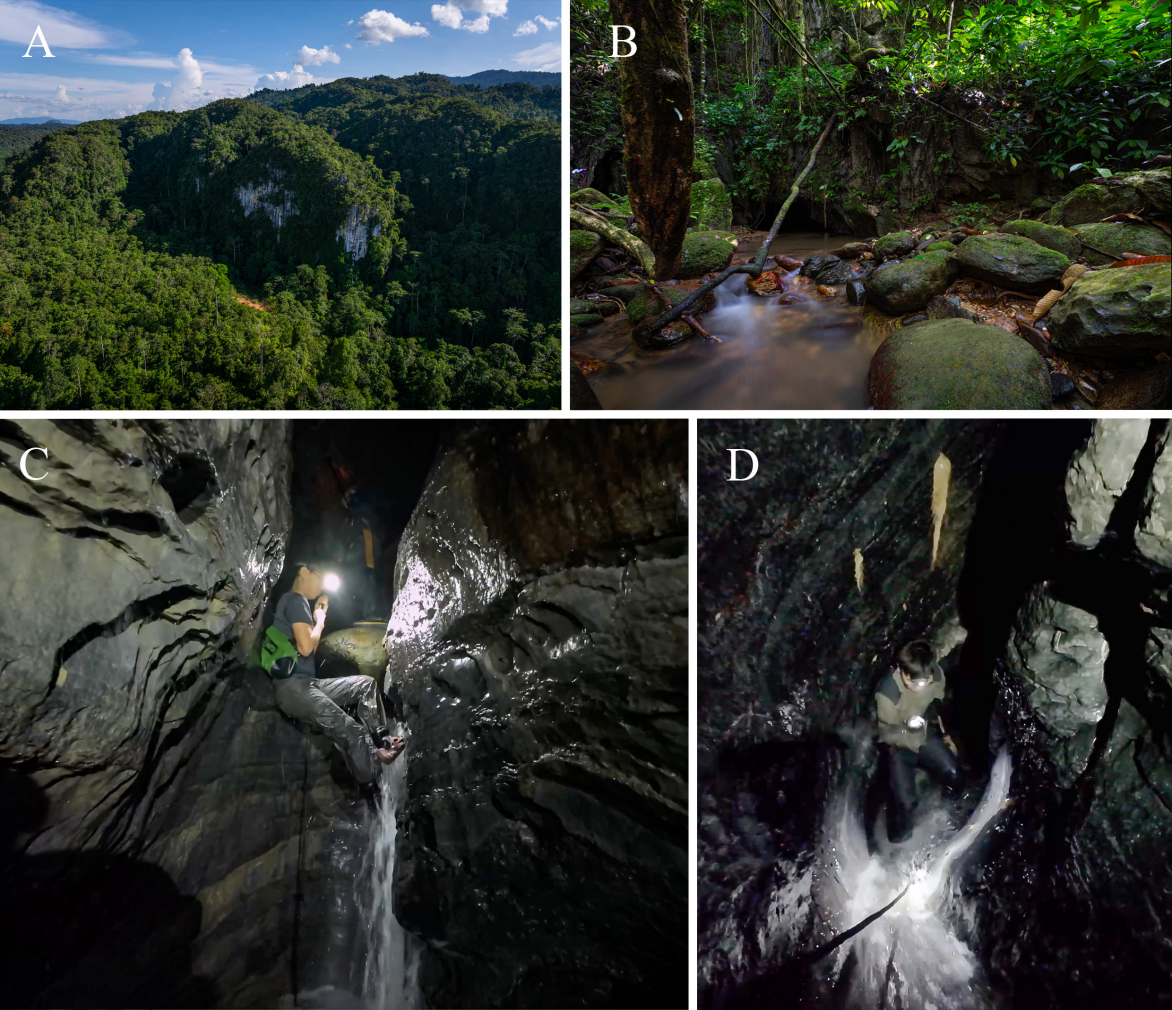
Type locality of *Merapohra karsticola* gen. nov. et sp. nov. Gua Hari Malaysia. **A**. Karst massif of Gua Hari Malaysia; **B**. Re-emergence of stream and entrance into Gua Hari Malaysia; **C**. Waterfalls in Gua Hari Malaysia; **D**. Narrow cave passage of Gua Hari Malaysia. (Photo credit: Mr. Siaw Yu Zhang).

*Merapohra karsticola* sp. nov., is so far only known from the type locality, Gua Hari Malaysia, Merapoh, Pahang, Peninsular Malaysia (Figs 1, 8).

## Discussion

Only two species of freshwater crab have been described from caves in Peninsular Malaysia thus far, *Phricotelphusa hymieri* and *Johora gua*. Both species are only known from their respective cave habitat. Similarly, *Merapohra karsticola* gen. nov. et sp. nov., has so far only been collected from Gua Hari Malaysia, a long narrow epiphreatic cave with sections featuring active speleothem. Within the cave, a constant fast flowing stream runs through, with several steep sections and waterfalls (Fig. 8C, D). The stream enters the cave passage on one side of the limestone massif forming a long narrow cavern passage (~ 880 m long), before re-emerging as a forest stream (Fig. 8B–D). In certain sections, the cave ceiling is low, just enough for a person to crawl through, while in other sections, the roof can extend for more than 10 metres, where bats were observed to be roosting. Due to the constant fast flowing stream, which sometimes rises significantly due to rainstorms, there is relatively little detritus or guano deposited in the cave, and the cave remains moist and cool. During our visit, the crabs were observed near holes or crevices, out of water, and usually near wherever little deposits of guano were present. Anecdotal accounts from local villagers suggest that the crabs are more frequently found on higher ledges of the cave, perhaps where guano deposits are more abundant, making the collection of this species challenging. Other inhabitants in the cave includes isopods, tailless whip scorpions (Amblypygi), an aquatic crab species (*Johora cf. booliati* Ng, 2020), frogs (*Limnonectes* sp.), and three species of fish, viz., *Neolissochilus* sp., *Mastacembelus* sp., and *Glyptothorax schmidti* (Volz, 1904). With the exception of the isopods and tailless whip scorpions, the remaining taxa observed are most likely surface-dwelling species, and their presence in the cave is probably accidental or occasional.

Specimens of *Merapohra karsticola* were only collected within the cave passage of Gua Hari Malaysia, and it possesses certain morphological characters which aids in its cavernicolous habits, viz., its dolichopodous form for climbing, scrambling on cavern walls, and a relatively flat carapace for squeezing into cave crevices. The species, however, is most clearly not an obligate cave dweller. Notably, *Merapohra karsticola* still possesses large, pigmented eyes, and a vibrant purple carapace (Figs 6, 7). This contrasts with true troglobitic species which possesses reduced eyes, with the cornea often reduced or absent, and varying degrees of de-pigmentation, a phenomenon that has been suggested to be due to strong, directional selection and not a result of the accumulation of selectively neutral mutations (Klaus et al. 2013). Examples of troglobitic freshwater potamid crab species found in Southeast Asia includes *Erebusa calobates* Yeo & Ng, 1999 (central Laos), *Cerberusa caeca* Holthuis, 1979, *C. tipula* Holthuis, 1979 (Mulu National Park, Sarawak, Malaysia), *Johora gua* Yeo, 2001 (Pulau Tioman, Peninsular Malaysia), *Ngan mayla* Guinot & Rodríguez Moreno, 2024 (Lubang Gedung Cave, Merabu Karst, East Kalimantan, Indonesia), and *Schubartamon serenum* Guinot & Rodríguez Moreno, 2024 (Vang Vieng Karst, Vientiane province, central Laos) (see Guinot and Rodríguez Moreno 2024b, for detailed comparison of troglomorphic features between various troglobitic species). The habits of *Merapohra karsticola* are very likely more akin to those observed in *Tiwaripotamon* species, being primarily karst dwellers present within caves and on the surface of limestone hills (Shih and Do 2014; Do et al. 2016, 2017; Dang and Do 2025).

The karst systems within the Southeast Asia have been shown to host rich diversity of endemic freshwater crabs (Ng and Ngo 2022; Promdam et al. 2022; Guinot and Rodríguez Moreno 2024a, 2024b; Dang and Do 2025), plants (Chin 1977; Kiew et al. 2014, 2017, 2019; Tan et al. 2024), herpetofauna (Orlov et al. 2004; Grismer et al. 2013, 2014, 2016, 2021; Poyarkov et al. 2018; Quah et al. 2021), mammals (Latinne et al. 2013; Hasrizal Fuad et al. 2024), and more. Despite their importance in harbouring a disproportionately high number of endemic species, karst systems in Peninsular Malaysia have been heavily exploited by man, e.g., for ore mining, cement quarrying (e.g., Gua Kanthan in Perak and Chiku in Kelantan) or in some cases become completely inundated by the construction of hydroelectric dams (e.g., Nenggiri Valley, Kelantan) (Grismer et al. 2014; Kiew et al. 2014; Nordin et al. 2025). As such, karst systems in the region are some of the most imperilled habitats globally (Clements et al. 2006; Liew et al. 2016).

For now, the karst habitats in and around Merapoh (that Gua Hari Malaysia is part of), are not facing any immediate threat. Previously, there were plans for the karst hills around Merapoh to be quarried and levelled for a cement production plant, but following petition and consultation from local villagers, the state government of Pahang reversed the plan, opting instead to develop karst-related eco-tourism for the region (Cilisos.my 2017). More recently, the Pahang state government designated Lipis district (includes Merapoh) as a geopark, highlighting the region’s geological significance (Hasrizal Fuad et al. 2024). It must be noted that the Geopark designation does not confer direct legal protection status in Malaysia unless the karst systems are part of a designated national park or forest reserve (Hasrizal Fuad et al. 2024). Moreover, studies have shown that destructive anthropogenic activities such as quarrying on limestone hills have detrimental effects and have led to the extinction of plant and animal species in Malaysia (Kiew 1991; Schilthuizen et al. 2005).

Freshwater crabs, in most cases, have a narrow distribution range or can even be hyper-endemics, and as such, are at high risk of extinction from anthropogenic activities (Cumberlidge et al. 2009). The low numbers of freshwater crabs known from karst systems or caves in Peninsular Malaysia are most likely an artefact of inadequate survey efforts, especially in comparison to the known diversity of karst/cave freshwater crabs from neighbouring countries (see Promdam et al. 2022). As such, there is an urgent need to conduct systematic and thorough biodiversity surveys at karst sites in Peninsular Malaysia as new, narrowly distributed, and endemic karst species could be lost even before they are discovered (Clements et al. 2006).

## Supplementary Material

XML Treatment for Merapohra

XML Treatment for Merapohra karsticola

## Additional information

### Use of AI

No use of AI was reported.

### Author ORCIDs

Zhi Wan Tan https://orcid.org/0000-0002-9878-6958

Qie Ooi https://orcid.org/0009-0001-1417-965X

Peter K. L. Ng https://orcid.org/0000-0001-5946-0608

### Data availability

All of the data that support the findings of this study are available in the main text.
